# Invasive Group A *Streptococcus* Outbreaks Associated with Home Healthcare, England, 2018–2019

**DOI:** 10.3201/eid2805.211497

**Published:** 2022-05

**Authors:** Laura E. Nabarro, Colin S. Brown, Sooria Balasegaram, Valérie Decraene, James Elston, Smita Kapadia, Pauline Harrington, Peter Hoffman, Rachel Mearkle, Bharat Patel, Derren Ready, Esther Robinson, Theresa Lamagni

**Affiliations:** Public Health England, London, UK

**Keywords:** Group A Streptococcus, Bacteria, Streptococci, Streptococcus pyogenes, public health, long-term care, infection control, disease outbreaks, whole-genome sequencing, wound infection, England

## Abstract

Healthcare-associated invasive group A *Streptococcus* (iGAS) outbreaks are common worldwide, but only England has reported outbreaks associated with home healthcare (HHC). We describe 10 outbreaks during 2018–2019 in England. A total of 96 iGAS cases (range 2–39 per outbreak) and 28 deaths (case-fatality rate 29%) occurred. Outbreak duration ranged from 3–517 days; median time between sequential cases was 20.5 days (range 1–225 days). Outbreak identification was difficult, but *emm* typing and whole-genome sequencing improved detection. Network analyses indicated multiple potential transmission routes. Screening of 366 HHC workers from 9 outbreaks identified group A *Streptococcus* carriage in just 1 worker. Outbreak control required multiple interventions, including improved infection control, equipment decontamination, and antimicrobial prophylaxis for staff. Transmission routes and effective interventions are not yet clear, and iGAS outbreaks likely are underrecognized. To improve patient safety and reduce deaths, public health agencies should be aware of HHC-associated iGAS.

## Abstract 2

*emm* typing and whole-genome sequencing can help identify case clusters.

*Streptococcus pyogenes* (group A *Streptococcus*; GAS) is a common community-acquired pathogen, predominantly affecting skin, soft tissues, and the respiratory tract. Invasive GAS (iGAS) infection, characterized by entry of the bacterium into sterile body fluids, including blood, has a mortality rate of 8%–16% (*1*–*4*). Person-to-person iGAS transmission is thought to occur through direct skin contact or via respiratory droplets from symptomatic infections and asymptomatic carriers. Throat, nose, skin, and anogenital carriage have been linked to healthcare-associated outbreaks (*5*–*8*), which have been recorded in hospital, long-term care, and outpatient facilities worldwide (*9*–*11*). Environmental and fomite transmission are less well characterized.

In England, most community nursing care is performed by practitioners traveling between patients to deliver healthcare in the patients’ homes, termed home healthcare (HHC). HHC is administered by a variety of healthcare workers, including district nurses, community nurses, healthcare assistants, general practitioners, podiatrists, hospital outreach teams, and palliative care staff. A substantial part of HHC is wound care, but HHC workers (HHCWs) also administer medication, assist with rehabilitation, and perform catheter and end of life care. During a single working week, an HHCW could perform many of these duties for different patients.

The home environment is not designed for healthcare and has unique infection control challenges. HHCWs and their equipment could become contaminated directly from the patient or the patient’s home, and the patient risks infection from practitioners or their equipment (*12*,*13*).

In England, iGAS cases are notifiable to local health protection teams (HPTs) under the Health Protection (Notification) Regulations 2010 (*14*) as a means of beginning immediate public health actions as needed, including contact tracing, according to national guidelines (*15*). Guidance also requests that all sterile site GAS isolates be sent for typing to the Respiratory and Vaccine Preventable Bacteria Reference Unit (RVPBRU) of Public Health England (PHE). All isolates, including GAS isolates from possible healthcare-associated infections, should be referred for typing or stored locally for future outbreak investigations. RVPBRU returns results to the referring hospital and local HPT within 6 days. RVPBRU also provides whole-genome sequencing (WGS) to support outbreak investigations.

In 2013, PHE identified the first HHC-associated iGAS outbreak in England (*16*). PHE has regularly recorded outbreaks since then, and HPTs managed outbreaks with advice from national leads for streptococcal surveillance and reference microbiology units. We describe HHC-associated iGAS outbreaks reported during January 2018–August 2019, including identification, investigation, and management, to inform public health response in England and elsewhere.

## Methods

### Case Definition and Data Sources

In this study, we included HHC-associated iGAS outbreaks identified in England during January 1, 2018–August 31, 2019. We identified outbreaks cross-referenced from PHE’s case and outbreak logging software, HPZone, and the RVPBRU streptococcal outbreak dataset. In addition, we contacted the healthcare-associated infection leads of each PHE center to identify any outbreaks not reported in the 2 datasets. We chose this short timeframe to ensure we could examine each outbreak in detail and maximize accurate data collection.

We included outbreaks with >2 cases of iGAS infection of the same *emm* type and linked to the same defined HHC service. We excluded outbreaks in which other exposures offered a more plausible transmission route, such as within residential care or another healthcare setting.

The inclusion criteria for individual cases within an outbreak varied between outbreaks and were set by the investigating outbreak control team (OCT). The broadest inclusion criterion for cases was defined as iGAS of the same *emm* type linked to the same defined HHC service. In outbreaks for which WGS was deployed, the inclusion criteria were honed to include only cases linked by sequencing, defined as <5 SNPs between strains. Noninvasive GAS infections and colonization were not systematically investigated or recorded in all outbreaks.

To investigate temporal trends in outbreaks, we also searched HPZone for outbreaks during January 1, 2013–December 31, 2017. We did not search other sources for outbreaks during this period and did not collect further data because the outbreaks were too distant in time for data to be accurate. We provide operational definitions used in this study (Table 1).

**Table 1:** Definitions used in a study of invasive group A *Streptococcus *infection associated with home healthcare, England, 2018–2019

Term	Definition
Invasive group A *Streptococcus* (iGAS) infection	Isolation of GAS from a normally sterile site, either by PCR or culture. For this study, iGAS also includes GAS infections in which GAS was isolated from a normally nonsterile site in combination with a severe clinical presentation, such as streptococcal toxic shock syndrome or necrotizing fasciitis
Group A *Streptococcus* (GAS) infection	Isolation of GAS from a non-sterile site in combination with clinical symptoms attributable to bacterial infection including fever (temperature >38°C), sore throat, wound infection, or cellulitis
Group A *Streptococcus* carriage	Isolation of GAS from a nonsterile site but no symptoms attributable to infection with this microorganism
Home healthcare (HHC)	Community health services, including district nursing teams, general practitioners, podiatry (chiropody), community midwifery, hospital outreach, and palliative care, which provide medical or nursing care within a patient’s home
Residential care	Live-in accommodation that provides 24-hour care and support to its residents

### Data Collection and Analysis

We conducted a 1-hour qualitative semistructured telephone interview with the chair of each OCT or other nominated staff member. We asked participants standardized open-ended questions grouped into themes surrounding outbreak identification, microbiology, investigation, and infection control. We encouraged participants to elaborate on answers by asking probing follow-up questions and incorporated themes that emerged in early interviews into subsequent interviews. We explored barriers to investigation and management in a similar way and encouraged participants to identify learning points and recommendations for future outbreaks. We collected data by using a standardized interview protocol and captured audio recordings of interviews to enable further review by the interviewer. We used thematic analysis to analyze qualitative data.

When available, we collected quantitative data regarding the number of HHCWs and patients screened and treated. We collected standardized pseudonymized data on case-patients, including age, iGAS onset date, hospitalization, and outcome. When sequencing was performed, we identified cases linked by sequence data (these data are not reported here). We recorded and analyzed data in Excel (Microsoft, https://www.microsoft.com) and Stata version 15 (StataCorp LLC, https://www.stata.com) and managed data in line with PHE’s information governance policy.

### Ethics Approval

This study was performed by PHE as part of its legal obligation to collect and process information about communicable disease surveillance and control under section 251 of the National Health Service Act 2006 (https://www.legislation.gov.uk/ukpga/2006/41/contents). No further ethics approval was required.

## Results

### Outbreak Characteristics

**Figure 1:**
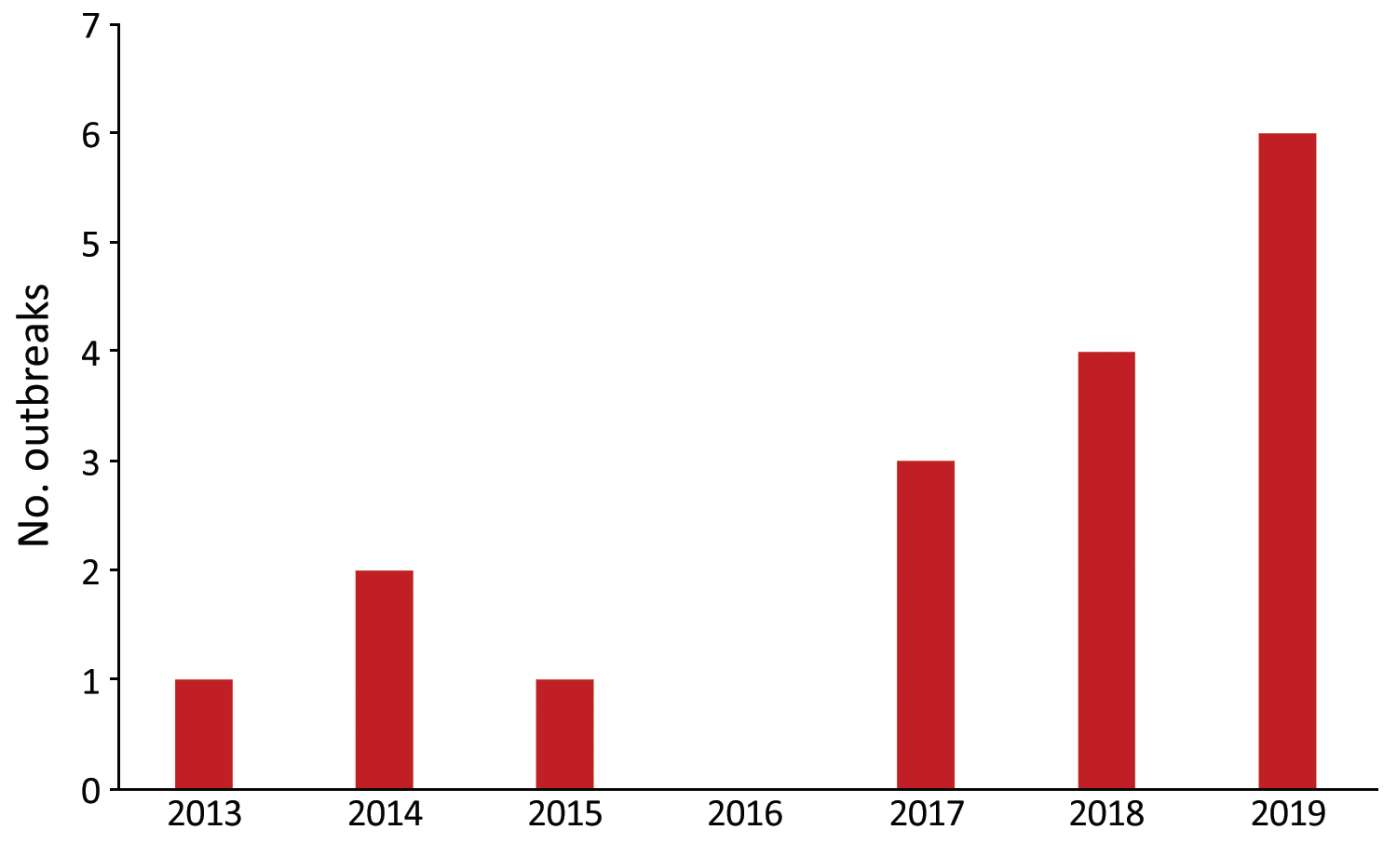
Annual number of home healthcare–associated invasive group A *Streptococcus* (iGAS) infection outbreaks reported to Public Health England, January 1, 2013–August 31, 2019. A total of 17 outbreaks occurred during this timeframe, but outbreaks sharply increased during 2018–2019.

During 2013–2017, a total of 7 HHC-associated iGAS outbreaks were identified in England; during January 1, 2018–August 23, 2019, a total of 10 HHC-associated iGAS outbreaks were identified (Figure 1). In these 10 outbreaks, 96 iGAS cases and 28 attributable deaths (case-fatality rate 29%) were reported. Outbreaks ranged from 2 to 39 (median 7) iGAS cases; case-level data and results of HHCW screening for 1 outbreak (outbreak number 10) were unavailable (Tables 2, 3).

**Table 2:** Summary of home healthcare–associated invasive group A *Streptococcus* infection outbreaks, England, 2018–2019*

Outbreak no.	No. iGAS cases	No. GAS cases†	No. deaths	No. days from first to last case	No. cases without identified HHC input	*emm* type	WGS
1	14	2	2	136	1	87	N
2	7	1	2	148	0	94	N
3	6	0	3	222	0	94	Y
4	7	0	2	388	0	89	Y
5	5	5	2	179	2	89	N
6	3	0	0	75	0	1	Y
7	4	0	0	219	0	1	Y
8	2	0	1	3	0	89	Y
9	9	1	1	507	0	89	Y
10	39	95	15	487	1	44	Y
Total	96	104	28	NA	4	NA	NA

*GAS, group A *Streptococcus*; HHC, home healthcare; iGAS, invasive group A *Streptococcus*; NA, not applicable; WGS, whole-genome sequencing. †Noninvasive GAS was not systematically investigated or recorded in all outbreaks. Available data did not enable distinction between carriage and noninvasive infection.

**Table 3:** Characteristics of home healthcare–associated invasive group A *Streptococcus* infection outbreaks, England, 2018–2019

Characteristics	No. (%)	IQR (range)
All outbreaks, n = 10		
Total cases	96 (100)	NA
Total deaths	28 (29)	NA
Median cases	7	4–9 (2–39)
Median outbreak duration, d	199	139–347 (3–507)
Outbreaks with case data, n = 9		
Case-patient characteristics, n = 57		
Median age, y	83	77–90 (42–100)
Sex		
F	39 (68)	NA
M	18 (32)	NA
Median days between cases	21	6–46 (1–225)
Type of residence, n = 48		
Residential care	17 (35)	NA
Own home	31 (65)	NA
HHCW exposure, n = 96		
Patient receiving care	92 (96)	NA
Household contact of recipient	2 (4)	NA
None identified†	2 (4)	NA

*HHCW, home healthcare worker; NA, not applicable. †Cases linked to outbreaks through whole-genome sequencing but without any identified connection to home healthcare services.

The median age of case-patients was 83 (range 42–100) years; 68% of cases were among female patients and 32% among male patients. Among 96 cases, 92 (96%) patients received nursing care administered by HHC services. Of the 4 cases that did not receive direct HHC care, 2 were household contacts of patients receiving HHC and neither had an identified GAS infection at the time. An epidemiologic link to HHC was not established for the other 2 cases, but those 2 were linked to other outbreaks by WGS.

Among 5 outbreaks with recorded wound swab sample results, GAS was cultured from 104 case-patients (range 1–95 cases per outbreak). The number of bacterial swab samples taken in these outbreaks was not documented by investigating teams, and available data did not enable distinction between GAS carriage and noninvasive infection (Table 2).

### Outbreak Identification

**Figure 2:**
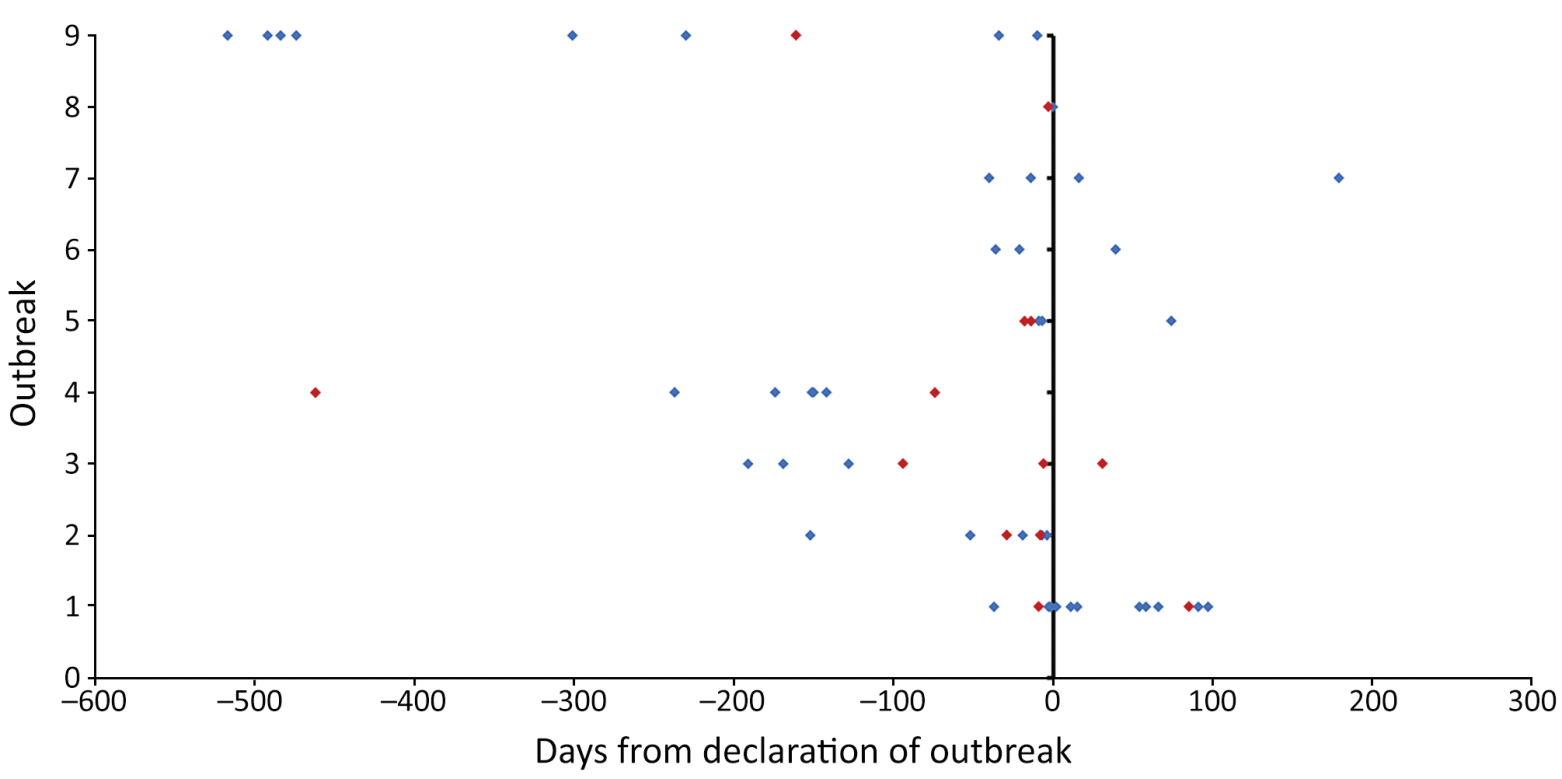
Timeline of cases in 9 home healthcare–associated invasive group A *Streptococcus* (iGAS) infection outbreaks, England, January 1, 2018–August 31, 2019. Vertical black line indicates date that outbreak was declared. Diamonds indicate day of initial detection of iGAS cases: blue diamonds represent patients that survived, red diamonds patients that died. Data from outbreak 10 (39 cases, 15 deaths) were not available.

Nine outbreaks were identified through statutory notifications of individual iGAS cases to local HPTs; 1 outbreak (outbreak 4) was identified through WGS at the RVPBRU Streptococcal Reference Laboratory. The median time between first identified case and the date the outbreak was declared was 40 days (range 3–517 days), but these data were not available for outbreak 10. Some cases were identified retrospectively when investigation teams reviewed previously notified iGAS cases of the same *emm* type to reinvestigate a link to HHC (Figure 2).

Six outbreaks were caused by *S. pyogenes* type *emm*1 or *emm*89, the 2 most common iGAS-causing *emm* types circulating in England during this period. Among the remaining 4 outbreaks, 2 were caused by *emm*94, 1 by *emm*87, and 1 by *emm*44. WGS was performed for 6 outbreaks involving *emm*1 (n = 2), *emm*89 (n = 3), and *emm*94 (n = 1) to establish whether cases of common *emm* types with epidemiologic links constituted an outbreak. Outbreak 10 (*emm*44) was sequenced because of the substantial number of cases and long duration (Table 2).

In the 6 outbreaks of common *emm* types (*emm*1, *emm*89, *emm*94), WGS confirmed that epidemiologically linked cases formed a genomic cluster in each outbreak. In 3 of these outbreaks, WGS identified >1 case of the same *emm* type with epidemiologic links to the outbreak that did not cluster with the other cases, enabling exclusion of the case from the outbreak. In 2 outbreaks, WGS confirmed that 2 sequential cases diagnosed >5 months apart but cared for by the same HHC team formed a genomic cluster and were likely part of the same outbreak. None of the sequenced outbreaks had close genomic relationships with each other, indicating each was a distinct outbreak.

One outbreak (outbreak 4) was not initially recognized by the local HPT but was identified by the reference laboratory from a set of local WGS controls used to investigate another HHC-associated iGAS outbreak (outbreak 9) (Table 2). The discovery of outbreak 4 revealed a separate *emm*89 iGAS in patients cared for by a single HHC team. Outbreak 4 involved 7 cases and 2 deaths over a period of 388 days, and the last case was notified 74 days before the outbreak was identified; no further cases were identified in the 60 days after the outbreak was identified. Although case-patients were cared for by a single HHC team, the epidemiologic link between cases was not identified earlier because the outbreak involved *emm*89, a common type; long intervals passed between sequential cases; and the HPT did not routinely ask about HHC exposures.

### Outbreak Duration

**Figure 3:**
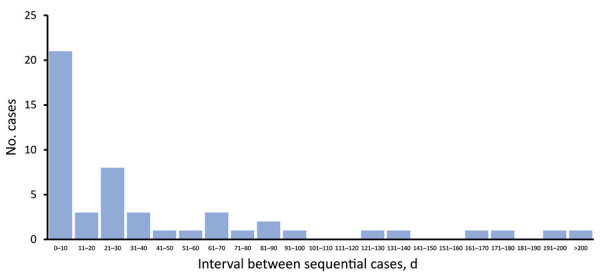
Intervals between sequential invasive group A *Streptococcus* (iGAS) cases in 9 home healthcare–associated outbreaks, England, January 1, 2018–August 31, 2019. Data from outbreak 10 were not available.

Duration of outbreaks varied greatly. The median time between specimen collection from the first and last identified case in each outbreak was 199 days (range 3–507 days). Long intervals often passed between cases (median 20.5 days, range 1–225 days) (Figures 2, 3).

In outbreaks 2, 4, 8, and 9, the last recognized case occurred before the outbreak was formally declared, and these outbreaks might have self-terminated after HHC teams instigated improved infection control and before the HPT became involved (Figure 2). Specifically, outbreaks 4 and 9 occurred in a region with a large concurrent HHC-associated iGAS outbreak in which HHC services had recently reviewed their infection control procedures. In the other 6 outbreaks, a median of 130 days (range 31–181 days) passed between outbreak declaration and the last identified case.

Once outbreaks were identified, time to link outbreaks to HHC was often delayed. Among 48 case-patients for whom place of residence was documented, 17 (35%) lived in residential care but also received HHC services. Transmission within the residential care facility initially was investigated before further cases were identified outside this environment and HHC links were explored.

### Outbreak Investigation

Investigating teams performed network analyses during outbreak investigations through records provided by HHC teams. These investigations did not identify a single HHCW in contact with all case-patients during the 7 days before symptom onset. HHCWs visited up to 20 patients per day, and multiple HHCWs might visit a patient each week, making investigation complex. In 5 outbreaks, >1 HHCW described symptoms suggestive of GAS before or during the associated iGAS outbreak. In addition, 8/10 OCTs reported difficulty obtaining information from HHC teams because of poor record keeping and time pressures on already overstretched services.

After network analyses, HHCWs were screened with throat swab samples for bacterial culture in all 10 outbreaks. The aim of screening was to identify HHCWs who might have acted as a common source and posed an ongoing risk to patients. In the 9 outbreaks for which data were available, a total of 411 HHCWs were identified for screening and 366 were screened by throat swab. A median of 22 (range 3–160) HHCWs were screened per outbreak. A single (0.36%) throat swab sample cultured GAS but unfortunately was not typed. In 7 outbreaks, any reported wounds or skin breaks among HHCWs were screened for GAS by swab and culture, but all were negative. In 3 outbreaks, a few HHCWs with negative throat swab samples but strong epidemiologic links to cases were screened with swab samples from piercing sites, perineum, and vagina; none were positive. The logistics of screening HHCWs in the community were complex, predominantly because of inadequate occupational health provision (6/8 outbreaks) and delays of up to 6 weeks between the decision to screen and commencement of screening. In addition, HHCW screening involved associated sensitivities, including concern about the use of screening to attribute blame and potential personal shame if swab samples were positive.

In 3 outbreaks, patient wounds were systematically screened for GAS carriage. In the 2 outbreaks with data available, 107 patients were screened but no GAS-positive samples identified. Although full data are not available for the third outbreak, GAS carriage and infection was detected in a small proportion of patients. In 7 outbreaks, patient wound screening was not systematically performed, but in 4 of these outbreaks HHCWs were encouraged to send swab samples from any wound with suspected infection. Although the number of swab samples sent for this indication is unknown, 6 swab samples from 2 outbreaks tested GAS-positive, but these were not *emm* typed, so they cannot be directly linked to other outbreaks.

In 2 outbreaks, environmental screening was performed. Bacterial swab samples were taken for culture from communal and storage areas at the HHCW base and from items that were difficult to clean, including portable electronic devices (e.g., tablets or smart phones), equipment, bags, blood pressure cuffs, and Doppler machines. Although the total number of swab samples taken was not recorded, a single swab sample taken from the handle of an equipment bag cultured GAS-positive, and subsequent WGS confirmed it to be the outbreak strain.

### Source and Transmission Mode

The sources and modes of transmission were not definitively established in any outbreak. The common hypothesis among investigating teams was that GAS was transmitted between colonized or infected patients and HHCWs and that numerous possible transmission events caused each outbreak. The role of fomites was unclear, but teams recognized the challenges associated with adequately decontaminating HHCW equipment in the home environment.

### Infection Control Methods

Infection control procedures were reviewed in each outbreak. Recommendations included infection control training for HHCWs and enhanced cleaning of HHCW bases and equipment storage areas in their cars. In 5 outbreaks, investigators noted that HHCWs carried equipment that was difficult to clean, such as fabric bags, portable electronic devices, and Doppler machines. This finding led to replacing fabric bags with impermeable, surface-wipeable bags (n = 3) or plastic, wipeable crates (n = 1), along with developing standard operating procedures for cleaning equipment that was difficult to decontaminate (n = 2). After outbreak 10 was identified, HHCWs were given disposable long aprons to wear during wound care procedures.

In 7 outbreaks, HHCWs were treated with antimicrobial drugs, which were intended to decolonize staff with potential occult carriage and interrupt transmission. In 6 outbreaks, HHCWs who had direct contact with a case-patient were initially treated with a 10-day course of penicillin V (median 2 [range 1–3] HHCWs per outbreak). When further cases occurred in 5 outbreaks, mass penicillin V prophylaxis for HHCWs was advised by the OCT and administered. In 4 outbreaks for which data were available, 139 HHCWs received prophylaxis (median 26 [range 22–65] per outbreak). In 3 of these outbreaks, no iGAS cases were notified after mass prophylaxis. HHCWs voiced opposition to antimicrobial drug prophylaxis in 3 outbreaks because of perceived lack of need after negative screening and concerns about antimicrobial resistance. In outbreak 1, the HPT directly engaged with HHCWs through presentations and discussions to achieve reasonable coverage and compliance with antimicrobial prophylaxis. Overall, HHCW compliance to antimicrobial prophylaxis is unknown.

Patients whose wounds cultured GAS-positive were treated with antimicrobial drug therapy. Mass antimicrobial prophylaxis was not administered to patients in any outbreak.

## Discussion

GAS outbreaks in hospitals, residential care facilities, and outpatient facilities are well documented, and guidelines exist for their investigation and management (*9*,*15*,*17*,*18*). However, despite a rising trend in HHC provision in Europe and the United States, the only published reports of HHC-associated iGAS outbreaks have come from England (*16*).

HHC-associated infections are common. Data from the United States suggest that 3.2% of HHC patients become infected and require hospitalization or emergency care treatment and that wound infections are among the most common (*13*). The home environment poses infection control challenges that differ from acute healthcare settings, including limited ability to decontaminate hands, equipment, and the environment, and a lower quality of environmental cleaning. In addition, family members who sometimes help nursing staff do not have adequate training in infection control. A recent study from Belgium highlighted the need for better data on HHC-associated infections and for developing infection control guidelines specific to this setting (*19*).

In England, the first HHC-associated iGAS outbreak was identified in 2013, and outbreak detection has been rapidly rising since then (*17*). Although all iGAS cases were notifiable in England during 2013–2021, characterization of isolates by the national reference laboratory is typically the trigger point for investigating clusters and no changes in isolate referral requirements were made during this period. However, local HPTs might have increasingly sought information on HHC after receiving advice from national teams, increased awareness, or both.

HHC services are under growing pressure because of a 46% reduction in qualified district nurses since 2010 and rising demand from an aging population with increasingly complex care needs. Nonspecialist nurses and healthcare assistants frequently are employed to deliver HHC. Among district nurses responding to a Queen’s Nursing Institute survey, 48% reported deferring visits or delaying patient care daily, 75% had unfilled vacancies on their teams, and 90% worked unpaid overtime hours (*20*). A King’s Fund report cited staff concerns over the quality and safety of care and reported wound care was particularly likely to be deprioritized during busy periods (*21*).

We noted substantial delays in outbreak identification; 1 outbreak in our study (outbreak 4) was only identified when sporadic case isolates were used as sequencing controls to investigate another outbreak. Although detection delays were polyfactorial, a major contributing factor was that most outbreaks were caused by the 2 most common *emm* types in England, *emm*1 and *emm*89, making it difficult to distinguish outbreaks from sporadic cases. Compounding this problem were long intervals, up to 7 months, between sequential cases and no standardized method for HPTs to record and review *emm* types. Although HPTs were mandated by national guidelines to inquire about previous hospitalization and residential care, they did not routinely ask about HCC.

The value of WGS in investigating iGAS outbreaks is becoming increasingly recognized. In this study, the increased discrimination of WGS over *emm* typing confirmed that epidemiologically linked cases of common *emm* types formed genomic clusters. WGS also identified epidemiologically linked cases that did not form genomic clusters with outbreak cases, enabling exclusion of cases from investigation. WGS identification of genomic case clusters focused outbreak investigations and management, particularly where complex HHC-associated cases had multiple common exposures, such as residential care, wound management teams, and podiatry. Routine and timely WGS of all iGAS isolates could result in early and accurate identification of outbreaks.

WGS findings highlight the complexities of GAS transmission within the community, including cryptic carriage and infection or fomite transmission as the most credible connection between genomic case clusters in patients with distant epidemiologic links. In this study, HHCW screening by throat swab with bacterial culture in 9 outbreaks identified only 1 GAS carrier. Possible reasons for this low detection rate include delays in instigating screening because of lack of occupational health support and resistance from HHCW, which might mean that GAS infection or carriage resolved before screening. In addition, some HHCWs swabbed themselves or their colleagues, which might have introduced bias resulting from concerns about attributing blame. Finally, most HHCWs were screened by throat swab alone, and multiple published outbreaks have shown that HHCW GAS carriage from other sites can be responsible for transmission. Negative throat swab samples should not be used to exclude infection in a HHCW with an epidemiologic link to cases (*16*,*18*).

GAS can persist on inanimate surfaces for up to 4 months and can contaminate fomites (*22*,*23*), but the role of fomites in GAS transmission is difficult to establish. Previous published outbreaks were attributed to a diverse range of sources, including showerheads and bed curtains, but these objects were not definitively established as the only GAS source (*17*,*24*). Because fomite surface contamination can be transient and superficial contamination can be readily lost via subsequent contacts, failure to find GAS on any specific item does not exonerate the item from the transmission pathway. In this study, a single swab sample from a fabric bag handle tested positive for GAS, but insufficient data were available on number of swabs taken, and insufficient environmental swab samples were taken in other outbreaks, to establish whether fomites were a common transmission pathway. However, this positive sample highlights that equipment and hand contact surfaces can become contaminated. All HHCW equipment should be easy to decontaminate between patients’ homes, and single-use equipment should be available where possible.

The first limitation of this study is that data were collected retrospectively and might have been subject to recall bias. No recommended guidelines on investigation of HHCW outbreaks were available when this study was performed, and OCTs did not have standardized data collection methods, resulting in missing data in some outbreaks. HHCW teams were not interviewed as part of this study and their insight on outbreak management would have been useful.

In conclusion, HHC-associated iGAS outbreaks are now common and increasingly recognized in England and have high mortality rates. Further work is needed to elaborate GAS transmission dynamics within the HHC environment and guidelines are required to guide HPTs in the investigation and management of these outbreaks. Outbreak control is complex and can require multiple interventions, including improved infection control, equipment decontamination, and prophylactic antimicrobial drug therapy for staff. Nonetheless, public health agencies should be aware of HHC-associated iGAS. Although outbreaks can be difficult to identify among sporadic iGAS cases, prompt *emm* typing and WGS offer a means for timely recognition of case clusters. 
